# Spiritual Care Experiences of Nurses Working in Gynecology Clinic: A Qualitative Research

**DOI:** 10.1111/jep.70030

**Published:** 2025-02-17

**Authors:** Fatma Başaran, Merve Şahin, Hava Salık

**Affiliations:** ^1^ Department of Midwifery, Faculty of Health Sciences Ağrı İbrahim Çeçen University Ağrı Türkiye; ^2^ Bergama Necla‐Mithat Öztüre State Hospital İzmir Türkiye; ^3^ Department of Health Management, Faculty of Economics and Administrative Sciences Hakkari University Hakkari Türkiye

**Keywords:** gynecology, nurse, qualitative research, spiritual care

## Abstract

**Rationale:**

Pregnancy and childbirth are among the most important events in a woman's reproductive life. In this process with full of uncertainties, nurses who provide primary care to women play a key role. Spiritual care is an important part of holistic care and an indicator of quality of care.

**Aim:**

This research aims to thoroughly examine and describe the spiritual care experiences of nurses working in obstetrics and gynecology clinics specific to their clinics.

**Methods:**

The study was carried out based on the descriptive phenomenology type of phenomenology design, one of the qualitative research methods. The study group included nurses in gynecology clinics in different provinces and hospitals in Turkey. The criterion sampling method, one of the purposive sampling methods, was used, and 12 nurses comprised the sample size. A semi‐structured interview form was used to collect the data. In evaluating the obtained data, thematic content analysis and MAXQDA 22 software were used. The study was written based on the COREQ checklist.

**Results:**

The data obtained from the interviews were analyzed under six themes: the concept of spiritual care, spiritual care practices, factors complicating spiritual care, factors facilitating spiritual care, emotional effects of spiritual care, and recommendations on the best way to provide spiritual care.

**Conclusions:**

It is vital to train gynecology nurses in spiritual care, reduce their workload, and develop clinical practice guidelines. Increasing the awareness of obstetric nurses about the factors affecting their spiritual care experiences while caring for individuals in clinical practices will contribute to increasing the quality of nursing care.

## 1 Introduction

Pregnancy and childbirth are among the most important events in a woman's reproductive life. During this period, women experience complex emotions such as happiness, faith, hope, anxiety, and fear at the same time. In this process with full of uncertainties, obstetrics and gynecology nurses who provide primary care to women play a key role [1]. Considering that pregnancy and birth are some of the most stressful life events in every woman's life, the nurse needs to assess the factors required to cope with the woman's stress and make it an enjoyable event. In recent years, it has been emphasized that pregnancy and birth are considered as a spiritual experience, and spiritual care is necessary to prepare women for birth [2].

Spiritual care is defined as the type of care provided to meet the patient's religious and existential needs regarding the meaning and purpose of life [3]. In 1998, the World Health Organization recognized spiritual health as the fourth dimension of health after physical, psychological, and social health to meet the needs of patients [4]. Moreover, the importance of meeting the spiritual care needs of patients has been emphasized internationally and included in various care guidelines [5]. Spiritual care is an important part of holistic care and an indicator of quality of care. Unmet spiritual needs lead to spiritual distress such as loneliness, decreased quality of life, hopelessness, decreased sense of spiritual peace, and depression [5, 6, 7].

Patient‐centered spiritual care should promote human contact through a compassionate relationship that encourages hope and comfort expressed through beliefs, values, traditions, and practices [7]. Nurses, who play an important role in patients' spiritual care, positively impact patients in spiritual care to improve their psychological coping skills, satisfaction, inner spiritual strength, and quality of life [8]. ^v^The American Nursing Association emphasized that the spiritual care competencies of nurses should be evaluated [9]. Nurses' spiritual care competencies are affected by many factors, such as age and education. Internationally, nurses show a lack of training and mentoring in spiritual care, express their inadequacy in assessing and addressing the spiritual domain, and express uncertainty *about whether patients' spiritual care needs* are met [10]. In a cross‐sectional study conducted by Kiaei et al. (2015), nurses stated that their knowledge about spiritual care was limited, they felt inadequate in providing spiritual care, and they did not receive adequate training [11]. In particular, gynecology nurses, who play an essential role in maternal and neonatal health, need to have the necessary skills and professional competencies to include spirituality in nursing practices effectively and safely.

In the literature, there is a lack of research on the provision of care for the spiritual needs that gynecology nurses may encounter and how they provide care for the spiritual needs of service users from the perspective of gynecology nurses. In light of this information, this study aimed to investigate the spiritual care experiences of nurses working in the gynecology clinic.

## 2 Methods

### 2.1 Research Type, Location and Time

This qualitative research was conducted between April 2 and May 20, 2024, with nurses with at least 1 year of clinical experience in gynecology actively working in hospitals in different provinces of Turkey. The study was carried out using the descriptive phenomenology type of phenomenological design, one of the qualitative research methods. Phenomenological design is a research method that enables people to express their understanding, feelings, perspectives, and perceptions about a particular phenomenon or concept. It describes how they experience this phenomenon [12]. The primary purpose of phenomenology is to understand the essence of the object by reducing individual experiences about an event to a universal explanation [13, 14]. Creswell (2016) discusses phenomenological studies in two different groups: (1) hermeneutic (interpretive) and (2) empirical (experimental, descriptive, unbiased). Descriptive phenomenology “emphasizes the description of experiences by the researcher rather than interpreting lived experiences.” For this purpose, qualitative researchers define the phenomenon ‐with phenomenology,” and emphasize the descriptive type of phenomenology. In descriptive phenomenology studies, rather than the participants' experiences, the researcher defines these experiences [13]. This phenomenological study was conducted as suggested by Miller (2003) [15], and the spiritual care experiences of the nurses working in the gynecology clinic were descriptively handled holistically by questioning the nurses' experiences. The Consolidated Criteria for Reporting Qualitative Research (COREQ) checklist guided the study.

### 2.2 Population and Sample

The study population consisted of gynecology nurses working in government hospitals in Turkey. The study's sample size was determined by taking into account the qualitative research design, the selected sample's diversity, and the participants' statements. The inclusion criteria for the participants were voluntarily agreeing to participate in the study, working in the gynecology clinic for at least 1 year, and accepting the online interview.

The sample was determined using the criterion sampling method, one of the purposeful sampling methods. The purposive sampling method used in qualitative research enables the selecting of people who are suitable for the study and who can best answer the research questions. In this method, people who meet the necessary criteria or have specific characteristics are preferred [13]. Criterion sampling is based on examining the sample that meets the criteria determined by the researcher [14]. Without calculating the sample size, interviews continued until data saturation was reached, and the sample group was finalized with 12 nurses in this process.

### 2.3 Data Collection

The data collection process was conducted online between April 2 and May 20, 2024, by a researcher with a doctoral degree in obstetrics and gynecology nursing and the Registered Nurse/Assistant Professor (FB) title. The researcher has received training and courses on qualitative research methods and is experienced in qualitative approaches. The data consisted of a first part with seven questions to determine the socio‐demographic characteristics of the nurses and a second part with eight open‐ended questions about the spiritual care experiences of the nurses prepared in accordance with the scientific literature. Before collecting the data, a pilot test was conducted with two nurses, and these data were not included in the study. When the research reached 12 women nurses, the purpose of the study was achieved, and the data collection process was completed. The interviews were conducted online and recorded at the convenience and lonely of the nurses. Verbal consent was obtained from the participants at the beginning of the interviews. Each interview lasted approximately 30–35 min. On the day of the interviews, the audio recordings were analyzed and recorded by giving code numbers to the participants without their names and identity information.

### 2.4 Data Analysis

After the data obtained from the interviews were transcribed by the researchers (FB, MŞ, HS) through the Transcriptor program (https://app.transkriptor.com/dashboard), each researcher checked the compatibility of the texts with the audio recordings, and missing or incorrect data were edited at this time. The data were analyzed using the descriptive analysis method in MAXQDA 22, a computer‐aided qualitative data analysis software. Descriptive analysis presents the obtained data to the reader in a format organized and interpreted according to the previously determined conceptual framework or themes. The data analysis methods of content analysis and descriptive analysis are the same. Data can be organized according to the themes revealed by the research questions or presented by considering the questions or dimensions used in the interview and observation processes. In descriptive analysis, direct quotes are used more to reflect the statements of the individuals interviewed or observed strikingly [16, 17]. The data uploaded to the program's system were read multiple times to holistically understand the participants' experiences, and the researchers independently coded them (FB, MŞ, HS). The differences between the researchers were discussed, and the arrangements continued until a consensus was reached on the themes. The coded data were categorized, and the first codes were formed and then turned into themes. After obtaining an expert's opinion with qualitative research experience in the nursing field, the themes were edited until a common opinion was reached. The results were tabulated and presented as themes, sub‐themes, and codes (Table 2). In the finalized texts, participant numbers N1‐N12 were used instead of the real names of the nurses.

### 2.5 Validity and Reliability

Validity and reliability in qualitative research are ensured through internal validity, external validity, internal reliability, and external reliability methods [18]. For the internal validity of the research, expert review was utilized, the research was shared with an expert academician who had previously conducted qualitative research and was asked to evaluate the research as a whole in terms of its conceptual dimension, objectives, problem, method, data collection tool, data analysis, and reporting. The research was finalized by considering the expert review's suggestions. For the external validity of the research, the research process, what was done in this process, the research model, the study group, the data collection tool, the data collection process, and the analysis and interpretation of the data were given in detail. For the internal reliability of the study, direct quotations were made from the opinions of the nurses, coding was done separately by the researchers (FB, MŞ, HS), and the percentage of inter‐coder agreement was calculated. The researchers read the data transcripts separately, coded the participants' statements, and created a code list. The coding reliability was calculated using the inter‐coder similarity formula proposed by Miles and Huberman and determined to be 83% [19]. For the external reliability of the study, descriptive data of the nurses were presented, how the interviews and data were recorded, how the analysis was performed, and how the results were combined and presented were explained in detail.

### 2.6 Ethical Considerations

Ethics committee permission of Hakkari University Scientific Research and Publication Ethics Committee dated 18.03.2024 and numbered 2024/45 was obtained for the study. The nurses participating in the study were informed about the aims and design of the study, and their verbal consent was obtained. The informed consent of the participants was obtained online, and the study was conducted in accordance with the Declaration of Helsinki.

## 3 Results

All of the nurses who participated in our study were female and aged 25 years and over. More than half of the nurses (58.3%) were married and had children. The majority of the nurses (83.3%) had Bachelor's degrees. The professional experience of the nurses ranged between 2 and 35 years. Moreover, the majority of the nurses (83.3%) evaluated their economic status as good, while 66.6% evaluated their religious beliefs as moderate (Table 1).

**Table 1:** Distribution of sociodemographic characteristics of nurses (*n* = 12).

Participants	Age	Marital status	Number of children	Education status	Professional experience	Perception of economic status	Perception of religious belief
**N1**	38	Married	2	Bachelor's degree	12 years	Good	Moderate
**N2**	28	Married	1	Bachelor's degree	6 years	Good	Moderate
**N3**	25	Single	No	Bachelor's degree	2 years	Good	Moderate
**N4**	44	Married	3	High school	23 years	Good	Strong
**N5**	38	Married	3	Master's degree	13 years	Good	Moderate
**N6**	44	Married	2	Bachelor's degree	23 years	Good	Moderate
**N7**	35	Single	No	Bachelor's degree	10 years	Poor	Weak
**N8**	44	Married	2	Bachelor's degree	24 years	Good	Strong
**N9**	30	Single	No	Bachelor's degree	8 years	Good	Moderate
**N10**	26	Single	No	Bachelor's degree	8 years	Poor	Moderate
**N11**	26	Single	No	Bachelor's degree	4 years	Poor	Strong
**N12**	58	Married	2	Associate degree	35 years	Good	Moderate

The spiritual care experiences of the nurses participating in the present study were determined to have five main themes (Table 2).

**Table 2:** Themes, sub‐themes, and codes related to nurses' spiritual care experiences (*n* = 12).

Theme	Subtheme	Codes
Concept of *Spiritual Care*	The meaning attributed to spiritual care	Psychological support
		Communicate
		Reducing anxiety
		Reducing fear
		Approach with compassion
Spiritual *Care Practices*	Psychological support	Being there for you Appropriate approach
	Contact	Speech Listening Providing information Being friendly
	Social support	Talking to family members
Factors *Complicating Spiritual Care*	Patient‐related factors	Cultural differences
		Failure to communicate
		Agitation Violence
	Environmental factors	Lack of comfort
		Professional team members
	Factors related to the patient's relatives	Lack of information
		Failure to respect privacy
		Problems within the family
Factors *Facilitating Spiritual Care*	Individual factors	Empathy
		Sociocultural factors
	Occupational factors	Providing training
		Friendly approach
		Effective communication
Emotional *Effects of Spiritual Care*	Effects on nurses	Occupational satisfaction
		Happiness
		Qualification
		Peacefulness
	Effects on patients	Satisfaction
		Trust
		Relaxation
		Motivation
Recommendations on the best way to provide spiritual care	Recommendations for health workers	Education
		Being friendly
	Recommendations for patients	Education
		Social support
	Recommendations for hospital administration	Improvement of hospital physical conditions
		Increasing the number of nurses
		Improving nurse salaries
		Single maternity rooms

### 3.1 Theme 1: The Concept of Spiritual Care

Gynecology nurses expressed the theme of spiritual care concept in a single Subtheme as the meaning attributed to spiritual care.

#### 3.1.1 Subtheme 1: The Meaning Attributed to Spiritual Care

It was stated that nurses attributed very special and beautiful meanings to spiritual care and played an essential role in establishing more robust communication with the patient. It was noted that the meanings attributed have vital aspects such as psychological support, establishing strong communication, and reducing anxiety and fear. The statements of some of the participants are as follows:

When I think of spiritual care, I think of the communication between me and the patient. I think of being able to trust each other.

(N5)

When I think of spiritual care, psychological support comes to mind. Since patients are in pain, it is part of our job to give them psychological support, to explain that this situation can happen, to try to reduce their anxiety and fear, and to comfort them by telling them that this situation is normal. This is what we do…

(N8)

### 3.2 Theme 2: Spiritual Care Practices

Nurses expressed the theme of spiritual care practices in three sub‐themes: psychological support, communication, and social support. It is observed that nurses mostly exhibit spiritual care practices with behaviors such as talking, listening, being friendly, and making them feel that they are with them to psychologically comfort them by trying to empathize with them patients.

#### 3.2.1 Subtheme 1: Psychological Support

Almost all of the nurses stated that they provided psychological support to the patients by ‘being with’ (*n* **=** 10), using an ‘appropriate approach’ (*n* **=** 7), and performing spiritual care practices in this way. The statement of one of the participants is as follows:

In addition to treating patients, that is, medical support, we provide spiritual care by being with them. I don't know; we provide appropriate care to make them feel good and peaceful. The energy we get when we first meet them is also very important. I think these affect our spiritual care practices, and everything becomes easier. No matter how tired we are, we are with the patient more, and therefore, the time we allocate to the patient increases…

(H3)

#### 3.2.2 Subtheme 2: Communication

Nurses stated that communication is a strong factor in spiritual care practices and that they communicate mostly through speaking, listening, giving information, and being friendly. The sample participant statement is quoted below as follows:

First of all, while giving spiritual care, I see them as individuals and treat them as such. I don't know their ideas, where they lack, don't know, or are afraid; I don't know how much knowledge they have about the birth process. I start by measuring this first. I chat with them, talk to them, listen to them. Then I inform them. Thus, I help them to realize that birth is not something to be afraid of. Either way, after they have already taken care of the communication steps, they become brave enough to ask questions after a while…

(H4)

#### 3.2.3 Subtheme 3. Social Support

Nurses stated that talking with family members is a practical spiritual practice in providing patients with social support. The statement of one of the participants is as follows:

We provide spiritual care. My practices are more effective, especially in the postnatal period when I am postpartum. Very young pregnant women come here frequently. You know, women and children, and they cannot express themselves. I ensure that they receive family support and inform family members so they do not feel more alone. And that they may be at ease…

(H1)

### 3.3 Theme 3: Factors Complicating Spiritual Care

Nurses expressed the theme of complicating factors while providing spiritual care in three sub‐themes: factors related to the patient, environmental factors and factors related to the patient's relatives.

#### 3.3.1 Subtheme 1: Patient‐Related Factors

Nurses stated that the cultural differences of the patients, their inability to communicate, agitation, and the emergence of violence factor were the complicating factors related to the patients in spiritual care practices. The statements of some of the participants are as follows:

Communication is sometimes tricky; there are complex patients. They push us very hard in every sense. We can also be subjected to insults. As if being subjected to verbal violence is not enough, it can also lead to physical violence. It can actually lead to physical violence. We have seen many pregnant women biting, kicking, shouting, and verbally abusing us on the delivery table. These are the problematic side of the job, but they are also negative….

(N2)

Pregnant women are not all the same. They do not all come from the same culture. Turkey is, unfortunately, very undeveloped in this regard. We work devotedly, but sometimes it is not enough…

(N4)

It is difficult if the patient is not open to communication. I think it is difficult for them to express themselves. They cannot say some things open‐heartedly. Sometimes, we try hard, but it doesn't work. I think this is the most difficult part of the gynecology department. It is not something to be ashamed of. In fact, this is the most important thing for them, so why should they be ashamed? We understand and try to explain, but we definitely cannot communicate.

(N7)

#### 3.3.2 Subtheme 2: Environmental Factors

Nurses stated that having problems with team members in spiritual care practices and not providing a comfort environment are environmental factors that make spiritual care difficult. Examples of nurse quotations related to this are as follows:

There are times when I have a hard time on duty. There is no gynecologist or pediatrician outside working hours. Since many things are instantaneous, it can be a problem. It is a baby's life. It can take time for doctors to come from home to the hospital. A lot of time is lost. We get very stressed. During these periods, we cannot explain to some doctors the seriousness of the event, and we have problems…

(N2)

The physical conditions are really bad. The pregnant woman should be alone. There should not be another patient with her, and her comfort should be ensured. Physical conditions need to be improved. I think this is also a problem for state hospitals in Turkey. The physical conditions and circumstances are always inadequate, always partially met, etc…

(N6)

#### 3.3.3 Subtheme 3: Factors Related to the Patient's Relatives

It was stated that the lack of knowledge of the patient's relatives, not respecting the privacy of the woman, and domestic problems were the complicating factors related to the patient's relatives experienced by the nurses in spiritual care practices. The statements of some of the participants are as follows:

The patient comes with her mother‐in‐law. She looks her mother‐in‐law in the eye to do something. She even has her confirm what we say. Mothers‐in‐law have traditional knowledge, and we cannot tell them anything. In conclusion, we are training the mother in vain. I ask myself if this is what I worked for. We need to train the mothers‐in‐law first, but that is difficult.

(N1)

In‐laws are one of the factors that hinder us. If the patient comes with her mother‐in‐law, it is too hard. That process ruins us. It isn't nice if the mother‐in‐law interferes when we try to teach them. We don't want them to come. They interfere too much. Because the sense of privacy is also very important for us, our pregnant woman cannot feel comfortable with her mother‐in‐law or sister‐in‐law at that moment.

(N3)

### 3.4 Theme 3: Factors Facilitating Spiritual Care

Nurses expressed the theme of factors facilitating spiritual care in two sub‐themes: individual and occupational.

#### 3.4.1 Subtheme 1: Individual Factors

Nurses stated that empathy and sociocultural factors are individual factors that facilitate spiritual care. They said that approaching according to the sociocultural level of the patient and being in the female gender is a facilitating factor in empathizing. The statements of some of the participants are as follows:

First of all, because we are women and because we are women, the essential thing in our profession is to empathize and put ourselves in their place. If I were like this, how would I want to be treated? This is spiritual care. Unfortunately, because we empathize, it is based on our conscience…

(N10)

It is crucial for me to stay calm. I need to remain calm and patiently treat patients according to their education level. Some patients understand, and patients do not. In this situation, selecting our words and expressions may be necessary. We need to be with them to understand their language, sometimes to integrate with their dialects, sometimes with the words they say. Because we are not all from the same part of the country…

(N11)

#### 3.4.2 Subtheme 2: Occupational Factors

It was stated that reducing the pain, fear, or anxiety experienced by women during childbirth or gynecological practices by providing education and information can prevent the negative behaviors that nurses are exposed to by women in these clinics, facilitate spiritual care practices, and that effective communication and being friendly are essential factors. The statements of some of the participants are as follows:

There are mums like that who attack here and there in labor; they want to throw themselves out. If we say whatever we want, it won't work. During the pandemic period, there were even people who spat in our faces. Amniotic fluid and saliva are coming. What have we seen? I'm sorry, but we work in filth. In fact, yes, we help in childbirth, but they need to be aware of this, so education is critical here. The education of the patients is also vital. We are university graduates, and everything is much easier when we are educated and provide training. Unfortunately, not every birth is the same, and not every pregnant woman's educational status is the same. Every birth is entirely different.

(N1)

…Spiritual care is a bit more humane. A humane approach is important for me because the other person is a human being. I care about them. I say hello or say how are you, and I approach them with a smiling face.

(N12)

### 3.5 Theme 4: Emotional Effects of Spiritual Care

Nurses expressed the theme of emotional effects of spiritual care in two sub‐themes: effects on nurses and effects on patients.

#### 3.5.1 Subtheme 1: Effects on Nurses

Nurses felt happy, competent, and peaceful and provided professional satisfaction with spiritual care practices. It is thought that spiritual care practices provide multidimensional positive emotions in nurses and that they perform their profession more fondly. The statements of some of the participants are as follows:

We can say that I am conscientiously comfortable and peaceful. I feel satisfied.

(N9)

Motherhood always makes you feel inadequate no matter what you do. Therefore, I need to provide the best education and support to pregnant women as much as possible. So don't feel inadequate. I feel better, I feel more competent, I think I fulfill my profession adequately, I feel happier when I get positive patient feedback.

(N2)

#### 3.5.2 Subtheme 2: Effect on Patients

Nurses stated that the spiritual care they applied had many positive emotional effects on patients, such as satisfaction, trust, relaxation, and motivation. It is seen in the quotations that these positive emotional effects on patients also positively affect nurses. The statements of some of the participants are as follows:

Once we have established a sense of trust, our patients and mothers can come here and say anything. They can ask everything they want to ask and learn everything they want to learn. They relax and communicate. Thus, the satisfaction of pregnant women and mothers increases.

(N1)

Mothers and pregnant women are motivated. I feel so peaceful. I am happy. God willing, with their prayers, we will perform this profession for many more years.

(N4)

### 3.6 Theme 5: Recommendations on the Best Way to Provide Spiritual Care

Nurses expressed the theme of recommendations for providing spiritual care in the best way in three sub‐themes: recommendations for healthcare professionals, patients, and hospital administration.

#### 3.6.1 Subtheme 1: Recommendations for Health Workers

It was stated that training on spiritual care practices, smiling, and strong communication and counseling skills can be effective practices for healthcare professionals in providing spiritual care practices. The statements of some of the participants are as follows:

I think we should be given updated training to have counseling skills. We should also be trained to improve ourselves. Communication and counseling skills are important. It should be applied to all staff.

(N5)

…I think the most important thing is to smile. If we want to support the other person, we need to smile. So, we need to walk around with our mouths in our ears. Smiling is really contagious.

(N7)

#### 3.6.2 Subtheme 2: Recommendations for Patients

It was stated that educating patients and their relatives, informing them through pregnancy schools, and having sufficient social support for the woman in this process would significantly contribute to the best delivery of spiritual care practices. The statements of some of the participants are as follows:

It should be ensured that women come and receive conscious education before the birth process. Apart from that, as I said, they can visit pregnancy schools and receive pregnancy training there…

(N3)

We can also interview their spouses or people in the family environment because they don't get support from their partners. They can be provided with social support. They hear a lot of negative things from their environment. Mothers or mothers‐in‐law criticize them about baby nutrition and physical conditions. These situations continue during the puerperium. Mothers‐in‐law and mothers can also be trained if the conditions are appropriate…

(N2)

#### 3.6.3 Subtheme 3: Recommendations for Hospital Administration

Nurses stated that hospital management has essential duties in addition to nurses, patients, and their relatives to ensure that spiritual care is provided best. It was said that providing a suitable physical environment, increasing the number of experienced midwives/nurses, improving salaries, and creating single delivery rooms by considering privacy would increase the quality of spiritual care. The statements of some of the participants are as follows:

…Our physical conditions should be good. Our physical conditions are not good. It is the problem with state hospitals. As such, everything is more restricted. We cannot ensure the privacy of the baby and the mother. This is a very comfortable thing at the same time. As I said, single delivery rooms should be available in all state hospitals. It needs to be fulfilled…

(N12)

The number of experienced nurses and midwives should be increased. Now, it's up to our administrators to fix it.

(N4)

## 4 Discussion

This study examined the spiritual care experiences of nurses working in a gynecology clinic. As a result of the analysis of the interviews, five main themes were identified: “the concept of spiritual care”, “factors complicating spiritual care “, “factors facilitating spiritual care”, “emotional effects of spiritual care”, and “recommendations on the best way to provide spiritual care”.

The only subtheme of the main theme of the concept of spiritual care was determined as “the meaning attributed to spiritual care”. Participants attributed many positive meanings (such as psychological support, communication, reducing fear and anxiety, and compassion) to spiritual care. In a mixed‐method study conducted with 282 nurses in South Korea, researchers expressed the meaning nurses attributed to spiritual care as four themes (helping to prepare for an honorable death with religious support, providing comfort and empathy, supporting spiritual satisfaction by finding meaning, and providing comprehensive care to the patient and family) [3]. In a quasi‐experimental study conducted with nurses, Riahi et al. (2018) stated that improving spiritual care provided by nurses can result in various outcomes such as increased patient satisfaction, decreased symptoms of anxiety and depression during hospitalization, shorter hospital stays, and overall improved quality of life [20]. As a result of studies conducted in societies and cultures with different religious beliefs, it has been concluded that nurses attribute positive meanings to spiritual care, and it benefits patients and their relatives.

The sub‐themes of the main theme of factors that make spiritual care difficult were determined as “patient‐related”, “environmental” and “patient relatives‐related” factors. It was determined that nurses had difficulty in communicating with patients, and factors such as cultural differences, lack of knowledge, and privacy made spiritual care difficult. Similar to the findings of previous studies, it has been determined that the most frequently mentioned barriers to providing spiritual care were lack of time/intense work tempo, insufficient knowledge and training on spiritual care, inability to communicate, diversity of spiritual needs of patients, and lack of privacy, respectively [3, 11, 20, 21]. It is thought that the excessive workload of nurses is an essential obstacle in providing spiritual care to patients, and nurses are inadequate in meeting the spiritual needs of patients because they cannot access sufficient information and training on this subject. Patient and nurse satisfaction can be increased by reducing the workload of nurses and organizing spiritual care training programs for health personnel. The absence of spiritual care units in our country and inadequate training are also essential factors. Establishing comprehensive spiritual care units in hospitals and integrating these units into other services over time by increasing the quality and number of training provided will positively contribute to both nurses and patients.

The sub‐themes of the main theme of factors facilitating spiritual care were determined as “individual” and “professional” factors. Factors such as empathy, training, effective communication, and a friendly approach were determined to facilitate spiritual care. In a cross‐sectional study conducted by Han et al. (2023) with nurses, it was determined that there was a significant relationship between nurses' personality characteristics and spiritual care behaviors [22]. It was reported that nurses' spiritual care training, long‐term working experience, and high level of education were effective factors in spiritual care competence and experience in providing spiritual care It is estimated that education is an important factor in spiritual care, positively affects the personality traits of nurses, and contributes positively to patient communication and satisfaction.

The sub‐themes of the main theme of emotional effects of spiritual care were determined as the effects on “nurses” and “patients”. It was determined that spiritual care contributed to professional satisfaction, qualification, happiness, and peacefulness in nurses; and satisfaction, trust, relaxation, and motivation in patients. In a study conducted by Güner and Akyüz (2023), it was stated that there was a significant positive relationship between nurses' spiritual levels and quality of care behavior and life satisfaction, and between spiritual care behaviors and life satisfaction [23]. There was a lack of studies on the emotional effects of spiritual care in different cultures and countries with religious beliefs. However, our findings are similar to the results of the study conducted in Turkey, a Muslim country, and it was concluded that spiritual care provides intrinsic motivation in both nurses and patients.

The sub‐themes of the main theme of suggestions for the best provision of spiritual care were determined as suggestions for “healthcare workers”, “patients” and “hospital administration”. The recommendations were determined as the healthcare professionals should be educated and friendly, the educational and social support of the patients should be increased, and the administration should increase the number of nurses, improve salaries, organize maternity rooms, and improve physical conditions. The results of previous studies are similar to our findings [3, 20, 22]. However, it is noteworthy that no previous study has been conducted directly with gynecology nurses. Although the workload of nurses is high, the low number of nurses and the lack of a satisfactory salary are factors that reduce satisfaction for both patients and nurses and reduce the quality of care.

## 5 Conclusion and Suggestions

In the present study, the spiritual care experiences of nurses working in the gynecology clinic were expressed as the meaning they attributed to spiritual care, facilitating and complicating factors, emotional effects, and suggestions. It was observed that nurses attributed positive meanings to spiritual care. However, they had high workloads, a lack of training and knowledge about spiritual care, problems with both patient and administration, and could not provide spiritual care completely. It is noteworthy that gynecology nurses, who play a key role for both mother and newborn, have an important gap in meeting patients' spiritual needs in nursing practices despite the interest in spiritual care in the clinic. This finding may guide nursing clinicians, educators, and policymakers to more effectively approach spiritual care as a useful component of holistic care. It is recommended to integrate spiritual content into educational programs and reducing the workload of nurses to enable more effective clinical delivery. Furthermore, it is thought that it would be useful to apply different cultural assessments to obtain more benefits from spiritual care practices.

### 5.1 Limitations

The data of this study are limited to the opinions of nurses working in gynecology clinics of state hospitals, and the results cannot be generalized. Future research should broaden the scope to understand different hospital staff better.

### 5.2 Implications to Practice

The study's findings provide a broad perspective on the problems experienced in gynecology clinics. By revealing the experiences of nurses who provide care and treatment to patients, it may be beneficial in eliminating problems, errors and difficulties in the knowledge, attitudes and skills of nurses working as health professionals regarding the care and treatment practices of individuals. Thus, it is thought to effectively protect the physical and mental health of nurses working professionally.

## Author Contributions

Study design: Fatma Başaran, Merve Şahin and Hava Salik. Data collection: Fatma Başaran. Data analysis: Fatma Başaran, Merve Şahin and Hava Salık. Study supervision: Fatma Başaran. Manuscript writing: Fatma Başaran, Merve Şahin and Hava Salık. Critical revisions for important intellectual content: Fatma Başaran, Merve Şahin and Hava Salık.

## Conflicts of Interest

The authors declare no conflicts of interest.

## Data Availability

The data that support the findings of this study are available from the corresponding author upon reasonable request.
